# A Mechanism of *O*-Demethylation of Aristolochic Acid I by Cytochromes P450 and Their Contributions to This Reaction in Human and Rat Livers: Experimental and Theoretical Approaches

**DOI:** 10.3390/ijms161126047

**Published:** 2015-11-18

**Authors:** Marie Stiborová, František Bárta, Kateřina Levová, Petr Hodek, Heinz H. Schmeiser, Volker M. Arlt, Václav Martínek

**Affiliations:** 1 Department of Biochemistry, Faculty of Science, Charles University, Albertov 2030, Prague 2 CZ-12843, Czech Republic; frantisek.barta@natur.cuni.cz (F.B.); katerina.levova@natur.cuni.cz (K.L.); hodek@natur.cuni.cz (P.H.); 2 Division of Radiopharmaceutical Chemistry, German Cancer Research Center (DKFZ), Im Neuenheimer Feld 280, Heidelberg 69120, Germany; h.schmeiser@dkfz-heidelberg.de; 3 Analytical and Environmental Sciences Division, MRC-PHE Centre for Environment and Health, King’s College London, London SE1 9NH, UK; volker.arlt@kcl.ac.uk

**Keywords:** plant nephrotoxin and carcinogen aristolochic acid I, cytochrome P450-mediated detoxification of aristolochic acid I, contribution of cytochromes P450 in detoxification of aristolochic acid I in human and rat livers, molecular modeling

## Abstract

Aristolochic acid I (AAI) is a plant alkaloid causing aristolochic acid nephropathy, Balkan endemic nephropathy and their associated urothelial malignancies. AAI is detoxified by cytochrome P450 (CYP)-mediated *O*-demethylation to 8-hydroxyaristolochic acid I (aristolochic acid Ia, AAIa). We previously investigated the efficiencies of human and rat CYPs in the presence of two other components of the mixed-functions-oxidase system, NADPH:CYP oxidoreductase and cytochrome *b*_5_, to oxidize AAI. Human and rat CYP1A are the major enzymes oxidizing AAI. Other CYPs such as CYP2C, 3A4, 2D6, 2E1, and 1B1, also form AAIa, but with much lower efficiency than CYP1A. Based on velocities of AAIa formation by examined CYPs and their expression levels in human and rat livers, here we determined the contributions of individual CYPs to AAI oxidation in these organs. Human CYP1A2 followed by CYP2C9, 3A4 and 1A1 were the major enzymes contributing to AAI oxidation in human liver, while CYP2C and 1A were most important in rat liver. We employed flexible *in silico* docking methods to explain the differences in AAI oxidation in the liver by human CYP1A1, 1A2, 2C9, and 3A4, the enzymes that all *O*-demethylate AAI, but with different effectiveness. We found that the binding orientations of the methoxy group of AAI in binding centers of the CYP enzymes and the energies of AAI binding to the CYP active sites dictate the efficiency of AAI oxidation. Our results indicate that utilization of experimental and theoretical methods is an appropriate study design to examine the CYP-catalyzed reaction mechanisms of AAI oxidation and contributions of human hepatic CYPs to this metabolism.

## 1. Introduction

**Figure 1:**
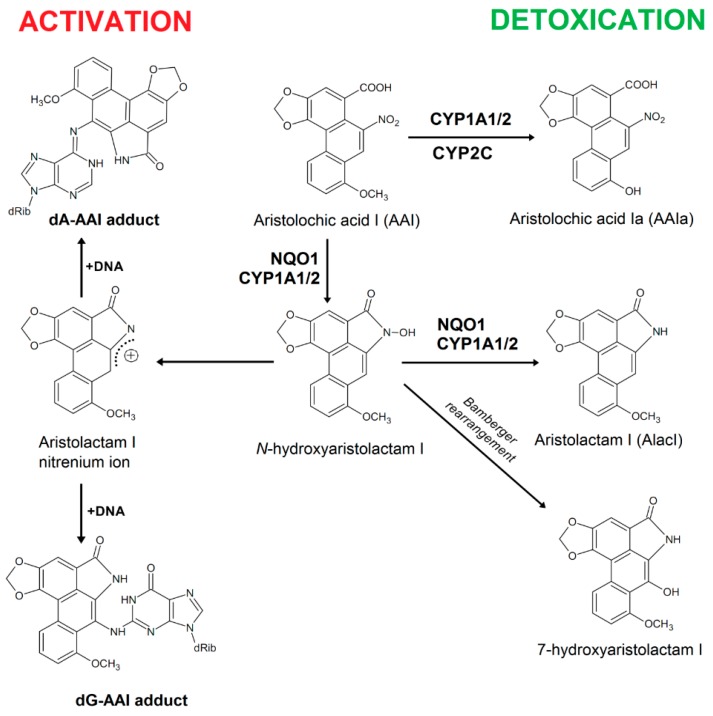
Activation and detoxification pathways of AAI. dA-AAI, 7-(deoxyadenosin-*N*^6^-yl)aristolactam I; dG-AAI, 7-(deoxyguanosin-*N*^2^-yl)aristolactam I; CYP1A1/2, cytochrome P450 1A1 and 1A2; CYP2C, cytochromes P450 of the 2C subfamily; and NQO1, NAD(P)H:quinone oxidoreductase.

Aristolochic acid (AA) is an herbal drug prepared from plants of the *Aristolochia* genus, where two alkaloids aristolochic acid I (AAI) (Figure 1) and AAII are the predominant chemical components [1]. Over twenty years ago, AA was shown to be the cause of a unique kidney disease Chinese herbs nephropathy (CHN), which is now assigned as aristolochic acid nephropathy (AAN) (reviewed in [1,2,3]). This specific renal fibrosis is associated with development of upper urothelial tract carcinoma (UUC) and, finally, bladder urothelial carcinoma [3,4,5,6]. AA is a Group I carcinogen as declared by the International Agency for Research on Cancer [7]. This plant alkaloid is also considered to participate in development of another kidney disease, Balkan endemic nephropathy (BEN), and its associated urothelial malignancy [8,9]. This disease is endemic in certain rural areas of Balkan countries which are localized closed to the tributaries of the Danube river basin [10].

In contrast to the findings that AAI might directly cause interstitial nephropathy, enzymatic activation of AAI to intermediates that bind to DNA is a necessary reaction leading to AA-mediated malignant transformation [8,9,11,12,13,14,15]. Indeed, exposure to AA has been proven by the detection of unique DNA adducts formed by AA in many tissues of patients suffering from AAN and BEN [9,10,16,17,18]. Specific AA-DNA adducts found in kidneys of patients are considered as biomarkers of exposure to AA; among them the 7-(deoxyadenosin-*N*^6^-yl)aristolactam I (dA-AAI) adduct is formed most frequently and is the long persistent adduct [1,5,10,18,19]. This DNA lesion produces specific A to T transversion mutations that were detected in the *TP53* tumor suppressor gene in tumors of patients suffering from AAN and BEN [8,9,20] and in immortalized Hupki (human *TP53* knock-in) mouse fibroblast cells (HUFs) treated with AAI [21], suggesting a molecular mechanism of AA-induced carcinogenic processes [8,22]. Interestingly, these A to T transversions have also been detected in other loci by whole-genome and exome sequencing analyzing AA-mediated UUC and AAI-exposed HUFs [23,24,25,26].

Nitroreduction of AAI, the compound that is considered as the major factor causing the AAN and BEN development, is required to exert its carcinogenic properties (*i.e.*, UUC development). Such nitroreduction results to the generation of *N*-hydroxyaristolactam I that leads to the formation of a cyclic acylnitrenium ion, the intermediate that either form DNA adducts or rearranges to 7-OH-aristolactam I (Figure 1) [2]. The product of AAI oxidation, 8-hydroxyaristolochic acid I (aristolochic acid Ia, AAIa), is a detoxification metabolite of this carcinogen. It is generated by *O*-demethylation of the methoxy group of AAI, and is excreted from organisms either in its free or conjugated forms [27,28,29,30] (Figure 1).

The concentration of AAI in organisms is crucial for both renal injury and induction of malignant transformations initiated by activated AAI. In addition to the quantities of AAI ingested by organisms, conversion of this chemical determines its actual concentration, thereby modulating also the clinical consequences of exposure. Hence, the characterization of enzymes that are mainly responsible for both detoxification and activation of AAI in humans as well as characterization of efficiencies of these enzymes in these reactions is of major importance.

Various enzymes metabolize AAI. Many studies demonstrated that NAD(P)H:quinone oxidoreductase (NQO1) acts as one of the most effective cytoplasmic nitroreductases reducing AAI to a cyclic acylnitrenium intermediate forming adducts in DNA [12,13,14,15,31,32,33,34,35,36,37]. In human and rodent liver microsomes AAI is reductively activated by cytochrome P450 (CYP) 1A2 and, to a lesser extent, by CYP1A1. Microsomal NADPH:CYP oxidoreductase (POR) also activates AAI in these organs [12,15,29,30,34,35,38,39,40,41,42,43,44,45]. However, CYP1A1 and 1A2 play a dual role in the metabolism of AAI. Whereas under anaerobic conditions they reductively activate AAI, under aerobic conditions these enzymes catalyze *O*-demethylation of the methoxy group of AAI forming AAIa (*i.e.*, detoxification) [12,13,14,15,29,30,34,38,39,40,41,42,43,46,47]. Recent studies have indicated that human and rodent CYPs of the 1A subfamily are the major enzymes oxidizing AAI under aerobic (*i.e.*, oxidative) conditions *in vitro* and *in vivo* (reviewed in [14,15]). Besides CYP1A/2, human and rat CYPs of the 2C subfamily also oxidize AAI [30,43,47,48] (Figure 1). The CYP-mediated AAI oxidation results to a decrease in AAI-mediated kidney injury [49,50].

However, there is still little information available about the impact of individual CYP enzymes on the oxidative AAI detoxification to AAIa in liver, the major organ for xenobiotic metabolism including AAI [30] in humans or animal models. Therefore, we evaluated contribution of individual CYP enzymes expressed in human liver to AAIa formation and compared it with that of CYPs expressed in livers of several animal models including rats that might, to some extent, mimic the fate of AAI in humans [35,48,51,52,53,54,55,56]. In order to compare efficiencies of hepatic microsomes of several species to oxidize AAI to AAIa, we previously analyzed generation of AAIa by human, rat, mouse, and rabbit liver microsomes [30,43]. The subcellular fractions from livers of all tested species were capable of catalyzing the oxidation of AAI to AAIa. The reaction was dependent on NADPH, a cofactor of POR-mediated CYP catalysis; without NADPH no oxidation of AAI was found. These results indicated that AAI oxidation by hepatic microsomes is mediated by CYP enzymes. Among the used microsomes, human and rat hepatic microsomes produced the most similar amounts of AAIa [30,43], indicating that rat hepatic microsomes seem to be an appropriate model to mimic oxidation of AAI in human hepatic microsomes. Therefore, human and rat enzymatic systems were utilized in this study.

## 2. Results and Discussion

To identify contributions of individual hepatic CYPs to AAI oxidation, three approaches were employed: (i) use of selective CYP enzyme inhibition in human and rat microsomes; (ii) use of human and rat recombinant CYPs; and (iii) analysis of the data on formation of AAIa by individual human and rat CYPs in these systems as well as those on the CYP enzyme expression levels in human and rat livers. Molecular modeling capable of evaluating interactions of AAI with the binding center of human CYPs was utilized to identify the molecular mechanisms of AAI *O*-demethylation catalyzed by human CYP enzymes that *O*-demethylate AAI in human liver.

### 2.1. Effect of CYP Enzyme Inhibitors on AAI O-Demethylation Catalyzed by Human and Rat Hepatic Microsomes

Under the experimental conditions used the individual CYP inhibitors were used in equimolar concentrations to that of AAI (10 μM). As shown in Table 1 and in our previous study [30], we found that AAIa formation in human microsomes was inhibited by α-napththoflavone, an inhibitor of CYP1A1/2, furafylline, an inhibitor of CYP1A2, and ketoconazole, an inhibitor of CYP3A, while inhibitors of other CYPs such as diamantane, an inhibitor of CYP2B, sulfaphenazole, an inhibitor of CYP2C, quinidine, an inhibitor of CYP2D, and diethyldithicarbamate (DDTC), an inhibitor of CYP2A and 2E1, were ineffective. In the present study we show that the same compounds inhibit AAIa formation also in rat hepatic microsomes, but in rat microsomes sulfaphenazole and DDTC also significantly decreased AAI oxidation (Table 1). The latter findings confirm that AAIa formation is catalyzed by hepatic CYP enzymes of both species and that human and rat CYP1A and 3A enzymes besides rat CYP2C, 2A, and 2E1 might be effective in the AAI *O*-demethylation reaction. These results also demonstrate a relatively weak potency of the CYP inhibitors under equimolar concentrations to that of AAI suggesting a high binding affinity of AAI to these CYP enzymes. However, although human and rat livers contained various CYPs, some of them are present in this human organ at limited amounts (*i.e.*, CYP1A1, 1B1, and 2B) [57,58]. Hence, this phenomenon may affect the degree of enzyme inhibition. Furthermore, it is important to mention that data found with inhibitors are sometimes difficult to be interpreted. Namely, the inhibitor can act more efficiently with one enzyme substrate than another.

**Table 1:** Effects of cytochrome P450 (CYP) inhibitors on AAI oxidation to aristolochic acid Ia (AAIa) by human and rat liver microsomes.

Inhibitor ^a^	AAIa Formation (% of Control without Inhibitor)	
	Human Microsomes	Rat Microsomes
α-Napththoflavone (CYP1A1/2)	89 ± 5 *	85 ± 5 **
Furafylline (CYP1A2)	75 ± 4 **	84 ± 5 **
Diamantane (CYP2B)	NI ^b^	NI
Sulfaphenazole (CYP2C)	NI	68 ± 3 ***
Quinidine (CYP2D)	NI	NI
DDTC (CYP2A, CYP2E1)	96 ± 5	52 ± 4 ***
Ketoconazole (CYP3A)	74 ± 4 **	90 ± 4 *

^a^ CYPs for compounds that act as their specific inhibitors are in brackets. Equimolar concentrations of individual inhibitors and AAI (10 μM) and 0.1 nmol of CYP were in incubation mixtures. The data are the mean ± SD of three parallel measurements (*n* = 3); ^b^ NI, no inhibition; *** *p* < 0.001, ** *p* < 0.01, * *p* < 0.05, statistically different from data of controls, without inhibitors (Student *t*-test).

### 2.2. O-Demethylation of AAI to AAIa by Human and Rat Recombinant CYPs in Supersomes™

**Figure 2:**
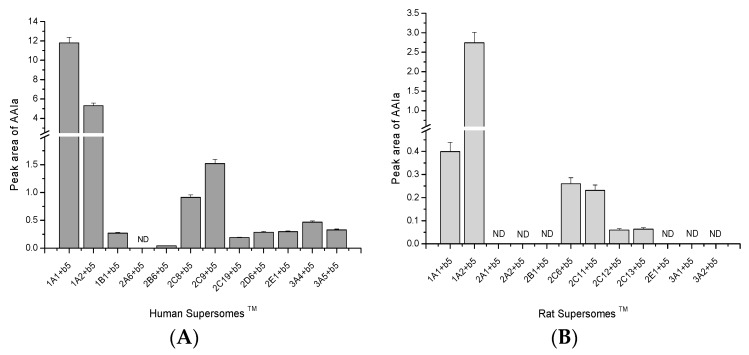
AAI *O*-demethylation to AAIa catalyzed with Supersomes™, each with a different human recombinant CYP (**A**) and rat recombinant CYP; (**B**) having these CYPs in combination with cytochrome *b*_5_ (b_5_). Data are averages ± SD of three parallel measurements (*n* = 3). ND, not detected. Data previously published in [30,41,43].

In former studies of our laboratory, we examined the activity of individual human and rat CYPs to oxidize AAI to its *O*-demethylation metabolite utilizing recombinant enzymes heterologously expressed in microsomal fractions of baculovirus-infected insect cells (Supersomes™) (Gentest Corp., Woburn, MI, USA) in combination with their reductase, POR [30,43]. However, this CYP system is not optimally corresponding to the natural microsomal system. In order to better mimic the situation in hepatic microsomes, individual CYPs were not only expressed with POR, but also with cytochrome *b*_5_, a known modulator of enzymatic activity of several CYPs [59,60,61,62,63,64,65,66,67,68,69,70,71,72]. We previously analyzed the efficiencies of human and rat CYP enzymes [30,41] to oxidize AAI to AAIa in the presence of this microsomal protein (Figure 2). In the experimental systems used, cytochrome *b*_5_ was either expressed in Supersomes*™* together with CYPs and POR or Supersomes*™* were reconstituted with purified cytochrome *b*_5_. Comparison of activities of individual CYPs to oxidize AAI to AAIa in the presence of cytochrome *b*_5_ with those determined previously without this microsomal protein [30,43] indicated that cytochrome *b*_5_ influences AAIa formation catalyzed by several CYPs. The strongest effect of cytochrome *b*_5_ was found on this reaction catalyzed by rat CYP1A1 and 1A2. The addition of cytochrome *b*_5_ to the incubation mixtures decreased AAIa formation catalyzed by rat CYP1A1, up to 50% relative to control (*p* < 0.001), and increased this reaction catalyzed by CYP1A2, by 1.2-fold (*p* < 0.01) (compare Figure 6 in [43] and data shown in Figure 2B). The potency of human CYP3A4 to oxidize AAI was also increased, by 1.4-times (*p* < 0.01) by cytochrome *b*_5_. In addition, an increase in AAIa formation catalyzed by CYP1A2 and 2C9 by cytochrome *b*_5_ was also found, but this increase was not significant [30].

Our results demonstrate that under the presence of the microsomal cytochrome *b*_5_ human CYPs are more effective in AAI oxidation than their rat orthologs. Human and rat CYP1A enzymes are the major enzymes oxidizing AAI. Other CYPs such as human and rat CYPs of the 2C subfamily and human CYP3A (CYP3A4/5), 2D6, 2E1, and 1B1, also form AAIa, but with much lower efficiency than CYP1A (Figure 2). For example, 7.9- and 3.5-times higher levels of AAIa were formed by human CYP1A1 and 1A2 than by the most efficient CYP enzymes of the 2C subfamily (*i.e.*, human CYP2C9), respectively. Likewise, human CYP1A1 and 1A2 were more than 13- and 5.8-fold more effective to oxidize AAI than another member of the CYP2C subfamily, human CYP2C8, respectively (Figure 2). Only rat CYP1A and 2C enzymes oxidize AAI of which CYP1A enzymes are more active than CYP2C enzymes (Figure 2B).

It should be emphasized that human/rat CYP1A1 and 1A2 orthologs show species-species differences in AAI preference, reaction velocities of its oxidation and the effects of cytochrome *b*_5_. Human CYP1A1 was found to be more effective to *O*-demethylate AAI than human CYP1A2, whereas rat CYP1A2 oxidizes this compound more efficiently than rat CYP1A1 (Figure 2).

### 2.3. Contributions of Individual CYPs to AAIa Formation in Human and Rat Livers

**Figure 3:**
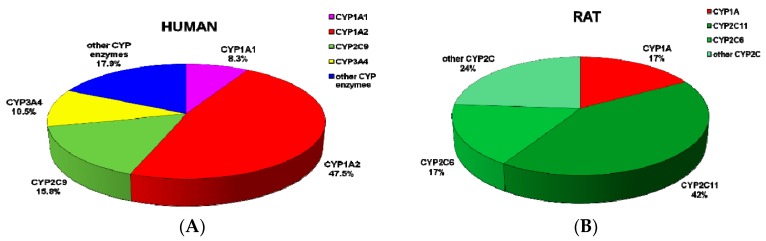
Contributions of CYP enzymes to AAIa formation in human (**A**); and rat livers (**B**).

Employing the results showing the velocities of AAI oxidation to AAIa by the Supersomal CYP enzyme systems containing cytochrome *b*_5_ (Figure 2) and the amounts of CYP enzymes expressed in human and rat livers [57,58,73,74,75,76,77,78,79,80,81,82,83], the contributions of individual CYPs to AAI oxidation in human and rat liver microsomes were evaluated. The highest contribution to AAI oxidation in human liver is attributed to CYP1A2 (~47.5%), followed by CYP2C9 (~15.8%), CYP3A4 (~10.5%), and CY1A1 (~8.3%). Even though the activity of human recombinant CYP1A1 to oxidize AAI is highest among all tested human CYPs (see Figure 2A), the low amounts of CYP1A1 in human livers (<0.7%) [75,83,84,85] caused that its contribution to the reaction in this human organ is lower than contributions of CYP1A2, 2C9 and 3A4 (Figure 3A). Of the other CYPs, CYP2E1, 2C8, and 2C19 also partially contributed to AAI oxidation, but only by ~1.1%, ~1.0% and ~0.6%, respectively. Contributions of other human CYPs (CYP1B1, 2B6, 2D6, and 3A5) in AAI oxidation in human livers are negligible.

In rat liver the highest contribution to AAI oxidation to AAIa is attributed to the CYP2C subfamily (~83%), mainly to CYP2C11 (~42%) and CYP2C6 (~17%), followed by CYP1A subfamily (~17%) (Figure 3B). Since the level of CYP1A1 expression in rat liver is around 10-fold lower than that of CYP1A2, the contribution of this CYP to AAI oxidation in rat liver is negligible (~1.7%).

### 2.4. Binding of AAI to the Active Sites of Compounds I of Human CYP1A1, 1A2, 2C9, and 3A4

**Figure 4:**
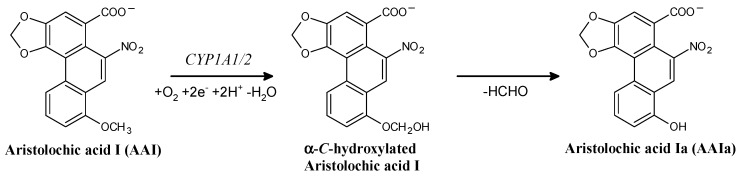
The CYP-mediated *O*-demethylation of AAI to AAIa.

*O*-Demethylation of AAI proceeds via the CYP-mediated attack of the carbon atom of the methoxy group by oxygen, which leads to the formation of the α-C-hydroxylation product that additionally decomposes to formaldehyde and AAIa (Figure 4). Therefore, the binding orientation of the methoxy group of AAI in the binary complex of AAI with the CYP active sites, which is a prerequisite process for *O*-demethylation of AAI, should dictate the efficiency of individual CYPs to catalyze this reaction. Thus, differences among abilities of the CYP enzymes to *O*-demethylate AAI (Figure 2) might be caused by the affinities of AAI to these enzymes and the binding orientation of the methoxy group of this compound in their active sites.

**Figure 5:**
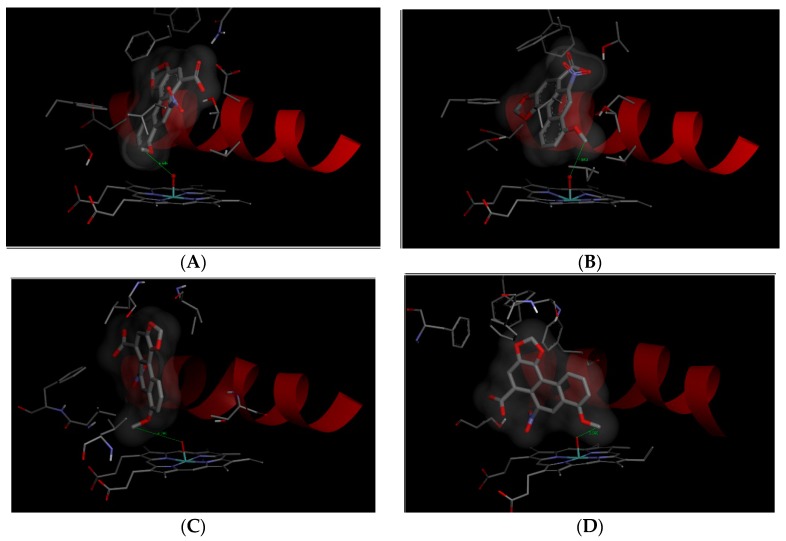
The binding orientations found in molecular docking calculations facilitating *O*-demethylation of AAI bound in human CYP1A1 (**A**); CYP1A2 (**B**); CYP2C9 (**C**); and CYP3A4 (**D**). AAI, heme and amino acids residues interacting ligand are rendered as bold sticks and sticks, respectively. Red ribbon represents a part of the I helix.

In order to investigate this suggestion, molecular modeling (*in silico* docking, employing soft-soft, flexible, docking procedure [84]) was used in this study. Using this method, we investigated binding of AAI to the active site of the Compounds I of human CYP1A1, 1A2, 2C9, and 3A4, the CYPs that all *O*-demethylate AAI but with different effectiveness and contribute efficiently to this reaction in human liver (Figure 5). The AAI molecule was successfully docked into the active sites of these CYPs. Every docking predicted several binding orientations of the AAI molecule. Positions showing short distances (3.7–4.9 Å) between the methoxy group of AAI and the activated oxygen atom in Compounds I of CYPs was found for all the CYP enzymes examined (Figure 5, Table 2). However, CYP2C9 and 3A4 enzymes are predicted to bind the AAI molecule with a significantly lower affinity than CYPs of the 1A subfamily (see values of free energies of binding shown in Table 2). The predicted binding free energy of AAI to human CYP1A1 is slightly lower than that to CYP1A2, but the distance between the carbon in the methoxy group of AAI and the oxygen atom on heme iron in the binary complex of CYP1A1 with AAI is shorter by 0.5 Å (Table 2). The larger distance of the reacting groups would result in a decreased reaction rate during CYP1A2-catalyzed AAI demethylation. Collectively, these results provide a suggestion why CYP1A1 and 1A2 are most efficient in AAI oxidation, while other CYPs (CYP2C9 and 3A4) are less active to catalyze this reaction (Figure 2A).

**Table 2:** The predicted binding free energies and distances facilitating *O*-demethylation of AAI bound in selected CYPs complexes.

Simulated System	The Most Stable Productive Orientations of AAI in the Complex with CYP	
	Estimated Free Energy of Binding (kcal/mol)	O(Comp I)-OCH_3_ (AAI) Distance [Å] ^a^
CYP1A1	−7.0	4.4
CYP1A2	−7.7	4.9
CYP2C9	−5.3	4.3
CYP3A4	−6.0	3.7

^a^ Distance between the carbon in the methoxy group of AAI and oxygen atom on heme iron in the complex of an activated CYP enzyme (Compound I) with AAI, see Figure 5.

## 3. Experimental Section

### 3.1. Supersomes™

Microsomes (Supersomes*™*) prepared from insect cells transfected with baculovirus constructs containing cDNA of single human CYP (CYP1A1, 1A2, 1B1, 2A6, 2B6, 2C8, 2C9, 2C19, 2D6, 2E1, 3A4, and 3A5) or their rat orthologs (CYP1A1, 1A2, 2A1, 2A2, 2B1, 2C6, 2C11, 2C12, 2C13, 2E1, 3A1, and 3A2), and expressing POR and cytochrome *b*_5_ were purchased from Gentest Corp. (Woburn, MI, USA). Supersomes™ containing rat CYP1A1/2 were reconstituted with cytochrome *b*_5_ (CYP: cytochrome *b*_5_, 1:5) isolated from rat liver microsomes by the procedures as described [80,85,86].

### 3.2. Preparation of Rat Hepatic Microsomes

Microsomal fractions were prepared from livers of ten male Wistar rats (AnLab, Prague, Czech Republic) by differential centrifugation as described previously [87].

### 3.3. Microsomal Incubations to Study AAI O-Demethylation

Incubation mixtures (250 μL) contained 100 mM potassium phosphate buffer (pH 7.4), 1 mM NADPH, 1 mg human or rat liver microsomal protein and 10 μM AAI [30,43]. Incubations were performed at 37 °C for 20 min; AAI oxidation (demethylation) to AAIa was determined to be linear up to 25 min. Control incubations were performed (i) without microsomal proteins; (ii) without NADPH; or (iii) without AAI. The optimum pH of the reaction mediated by human and rat liver microsomes was found to be pH 7.4; a decrease or an increase in pH to 6.4 and 8.4 lead to up to a 1.7- or 1.9-fold decrease in AAIa oxidation, respectively. This pH was therefore used in additional experiments. For Supersomes™, incubation mixtures (final volume 250 μL) consisted of 100 mM potassium phosphate buffer (pH 7.4), 1 mM NADP^+^, 10 mM MgCl_2_, 10 mM d-glucose 6-phosphate, 1 U/mL d-glucose 6-phosphate dehydrogenase (NADPH-generating system), 50 nM CYPs in Supersomes*™*, and 10 μM AAI. Supersomes*™* with POR alone were utilized as controls. AAI and its metabolites (*i.e.*, AAIa) were extracted from incubation mixtures with 2 × 1 mL of ethyl acetate and evaporated to dryness; residues were dissolved in 30 μL of methanol and analyzed with reverse-phase HPLC as described [43,46].

### 3.4. Inhibition Studies

Inhibition studies in human and rat liver microsomes were conducted similarly as shown previously [30]. α-Naphthoflavone (α-NF), which inhibits CYP1A1 and 1A2; furafylline, which inhibits CYP1A2; diamantane, which inhibits CYP2B; sulfaphenazole, which inhibits CYP2C; quinidine, which inhibits CYP2D; diethyldithiocarbamate (DDTC), which inhibits CYP2E1 and CYP2A; and ketoconazole (KC), which inhibits CYP3A, were employed to analyze inhibition of AAI oxidation by human and rat liver microsomes. Compounds were dissolved in 2.5 μL methanol (except for DDTC that was dissolved in distilled water) to yield final concentrations of 10 μM in the incubation mixtures. Inhibitors (10 μM) were incubated at 37 °C for 10 min with the NADPH-generating system prior to addition of 10 μM AAI, and then incubated for an additional 20 min at 37 °C. AAIa formation was analyzed by HPLC as described above.

### 3.5. Contributions of CYP Enzymes to O-Demethylation of AAI in Human and Rat Livers

In order to calculate the contributions of individual CYPs to AAI oxidation in human and rat livers, we utilized the velocities of AAI oxidation to AAIa by the Supersomal CYP enzyme systems containing cytochrome *b*_5_ (Figure 2) in the combination with the data on the expression levels of CYPs in human and rat livers [57,58,73,74]. The contributions of these enzymes were calculated by relative activity factor because the activities of CYP in Supersomes™ should be considered in addition to the relative contents of CYPs in the livers. Therefore, the contributions of each CYP that oxidizes AAI in livers were calculated by dividing of the relative activity of each of such CYPs oxidizing AAI [r.a._cypi_] (rate of AAI oxidation multiplied by amounts of this CYP in tissues examined), by the total relative activities (**∑**[r.a._cypi_]) of all CYPs oxidizing this substrate. Of human liver CYPs, CYP3A4 is the major enzyme present in this human organ (~30% of the CYP hepatic complement), followed by CYP2C9 and 1A2 (~15% and ~13%, respectively), while CYP2C19, 2E1 2A6, 2D6, 2C8, and 3A5 are present in human liver in levels of the range of ~8.5%–~2.5% of the liver CYPs (see [58] for an overview). CYPs of the 2C subfamily (CYP2C6, 2C11, 2C12, and 2C13) are the major enzymes expressed in rat livers accounting of ~55% of the CYP complement [57]. Of the CYP2C complement, ~50% and ~20% correspond to CYP2C11 and 2C6, respectively [73,74,83]. Of the other CYPs, CYP2E1, 3A, 2D, 2A, 2B, and 1A enzymes are also present in rat livers, expressed in levels of ~15%, ~10%, ~7%, ~6%, ~5%, and ~2%, respectively [57].

### 3.6. Molecular Docking of AAI into Compounds I of Human CYP1A1, 1A2, 2C9, and 3A4

The X-ray based coordinates of human CYP1A1 (2.6 Å resolution, PDB ID 4I8V) [88], human CYP1A2 (1.95 Å resolution, PDB ID 2HI4) [89], CYP2C9 (2.45 Å resolution, PDB ID 4NZ2), and CYP3A4 (2.74 Å resolution, PDB ID 1W0G) were used as starting structures for modeling of AAI interactions with the ground state of CYP enzymes. During structure preparation, hydrogen atoms were added and crystallographic water and ligand molecules were removed, usual protonation states and Gasteiger partial charges were assigned to all residues, except for the atomic charge of the ferric ion of the heme cofactor, for which a value more consistent with a metal in octahedral coordination was used [90]. The geometries and charges of a ligand (AAI) were predicted using *ab initio* calculations on the Hartree-Fock level of theory in conjunction with the basis set 6-31+G(d). All *ab initio* calculations were carried out with program Gaussian 03 [91].

We utilized a hybrid global-local Lamarckian genetic algorithm implemented in Autodock v4.2.6 program [84] suite to evaluate binding free energies and preferred binding modes for studied compounds. The Autodock v4 combines two procedures to find the most preferable binding modes, rapid grid-based energy evaluation and efficient search of torsional freedom, together with optional soft-soft docking. During the flexible docking procedure, both the position of the ligand and the orientations of the selected flexible side-chains are optimized simultaneously. In order to allow the enzyme to adapt to a new ligand, we ran soft-soft docking calculations. All rotatable bonds of the ligands and 10-11 selected amino acid side chains, CYP1A1 (S122, F123, N221, F224, F258, D313, D320, T321, V382, L496, T497), CYP1A2 (T124, F125, T223, F226, F260, D313, D320, T321, L382, L497, T498), CYP2C9 (V113, F114, I205, L208 T301, L366), and CYP3A4 (F108, S119, F213, F215, F241, F304) were allowed to rotate freely. We carried out an extensive search (2000 docking runs per system) of the most preferred binding modes of an AAI molecule within a 57 × 47 × 47 grid-box centered on the substrate binding cavity. Similar resulting structures (RMSD lower than 1.0 Å) were grouped and finally sorted by binding free energy of the best binding structure within each cluster. A set of binding modes with similar binding energies was found for every system as a result. We assume that only the orientations with a sufficiently short distance between carbon of the methoxy group of AAI and the activated oxygen atom in the CYP Compound I would facilitate the AAI oxidation.

### 3.7. Statistical Analyses

Statistical analyses were performed with Student’s *t*-test and *p* < 0.05 was considered significant.

## 4. Conclusions

The data presented in this study advance our knowledge on the oxidative detoxification of the human carcinogen AAI by human and rat CYPs and explain the differences in efficiency of human CYP1A1, 1A2, 2C9, and 3A4 enzymes to oxidize AAI. Human and rat CYP1A1 and 1A2 are the major enzymes oxidizing AAI. Other CYPs, such as human and rat CYPs of the 2C subfamily and human CYP3A4/5, 2D6, 2E1, and 1B1 also form AAIa but with much lower efficiency than CYP1A enzymes. Based on the amounts of AAIa formed by the tested human and rat CYP enzymes and the levels of CYP expression in human and rat livers, their contributions to AAIa formation in these organs were determined. The highest contribution to AAI oxidation in human liver is attributed to CYP1A2 (almost 50%), followed by CYP2C9, CYP3A4, and CYP1A1 (approximately 10%–15% each). In rat liver, the CYP2C subfamily contributes more than 80% to AAI oxidation, mainly CYP2C11 (roughly 40%) and CYP2C6, followed by CYP1A (nearly 20% each). The importance of these CYP enzymes to oxidize AAI in human and rat liver were confirmed by inhibition studies utilizing selective inhibitors of individual CYPs in hepatic microsomes of both species. These results are also in concordance with data found in *in vivo* studies utilizing *Cyp1a*/*Por-*knockout- or *CYP1A*-humanized mouse lines [15,29,30,42,43,47], as well as rat models [35,48] indicating the importance of human ad rodent CYP1A and 2C in AAI oxidation *in vivo*.

Flexible *in silico* docking modeling studies helped to understand the enzymatic differences in AAI oxidation by human CYP1A1, 1A2, 2C9, and 3A4 indicating that the binding orientations of the methoxy group of AAI in the CYP active centers and the energies of AAI binding dictate the efficiencies of these CYP enzymes in AAI oxidation. These results demonstrate that both the activities of individual human and rat CYPs and their expression levels in the liver dictate the degree of AAI detoxification in this organ. Therefore modulation of levels and activities of hepatic CYPs mediated by their polymorphisms or internal regulation, including their induction or inhibition by endogenous and exogenous compounds, determines AAI (geno)toxicity. The utilization of experimental and theoretical approaches is a useful tool to investigate the CYP-catalyzed reaction mechanisms, as demonstrated here for AAI.
